# Advancements in Ultrasound and Ultrasound-Based Risk Stratification Systems for the Assessment of Thyroid Nodule

**DOI:** 10.3390/cancers14071668

**Published:** 2022-03-25

**Authors:** Pierpaolo Trimboli

**Affiliations:** 1 Servizio di Endocrinologia e Diabetologia, Ente Ospedaliero Cantonale (EOC), 6500 Bellinzona, Switzerland; pierpaolo.trimboli@eoc.ch; 2 Facoltà di Scienze Biomediche, Università della Svizzera Italiana (USI), 6900 Lugano, Switzerland

Ultrasound (US) is an essential in-office imaging procedure used for evaluating thyroid nodules. This Special Issue entitled “Risk Stratification of Thyroid Nodule: From Ultrasound Features to TIRADS” published in *Cancers* allows us to improve the information about US and US-based risk stratification systems used for the assessment of thyroid nodules. Neck and thyroid US has been widely used during the last two to three decades and several significant developments have been reported in terms of the performance of US to detect thyroid cancer [1]. After an initial phase in which most clinicians used single US parameters in clinical practice, several international societies in the field of thyroid diseases have developed specific US-based systems (i.e., Thyroid Imaging Reporting And Data Systems, TIRADS) to improve the performance of US operators and standardize their terminology [2]. The latter represents a non-negligible advancement that eminent cytologists have also involved in the management of thyroid nodules [3]. Obviously, further efforts still are needed to achieve the optimal performance of US and TIRADSs, and the present Special Issue will contribute to these efforts. If how to discriminate benign from malignant lesions among the indeterminate nodules is still a matter of debate, the meta-analysis by Borowczyk et al. [4] reports interesting findings about the US differences between follicular adenoma and follicular carcinoma. The presence of Hashimoto’s thyroiditis is a potential pitfall when assessing thyroid nodules with US and the paper by Słowińska-Klencka et al. [5] analyzes the impact of changes in the threshold for the nodule’s shape criterion in four TIRADSs. Thermal ablation of benign thyroid nodules can represent another pitfall when we face previously treated patients and this was addressed by Bernardi et al. [6]. Other specific data have been reported about the role of contrast-enhanced US [7], grading of hypogenicity [8], assessment of neck lymph-nodes [9], and the potential future impacts of artificial intelligence on the thyroid field [10]. Moreover, how particular thyroid nodules, such as autonomously functioning nodules, may be put in the TIRADSs categories are reported by Seifert et al. [11]. Finally, the performance of TIRADSs in detecting thyroid cancer in a pediatric population was assessed by Scappaticcio et al. [12] and Piccardo et al. [13]. Overall, ultrasound is increasingly a necessary and essential tool in order to manage patients with thyroid nodules [14] and these new advancements can be useful in clinical practice.
